# The Representativeness of Participants With Severe Mental Illness in a Psychosocial Clinical Trial

**DOI:** 10.3389/fpsyt.2018.00654

**Published:** 2018-12-04

**Authors:** John Lally, Rochelle Watkins, Sarah Nash, Hitesh Shetty, Poonam Gardner-Sood, Shubulade Smith, Robin M. Murray, Fiona Gaughran

**Affiliations:** ^1^Department of Psychosis Studies, Institute of Psychiatry, Psychology & Neuroscience, King's College London, London, United Kingdom; ^2^Department of Psychiatry, Royal College of Surgeons in Ireland, Beaumont Hospital, Dublin, Ireland; ^3^Department of Psychiatry, School of Medicine and Medical Sciences, University College Dublin, Dublin, Ireland; ^4^St Vincent's Hospital Fairview, Dublin, Ireland; ^5^Biomedical Research Centre (BRC) Case Register, South London and Maudsley NHS Foundation Trust, London, United Kingdom; ^6^Department of Forensic and Neurodevelopmental Science, Institute of Psychiatry, Psychology and Neuroscience, Kings College London, London, United Kingdom; ^7^Forensic Intensive Care Service, South London and Maudsley NHS Foundation Trust, London, United Kingdom; ^8^National Psychosis Service, South London and Maudsley NHS Foundation Trust, London, United Kingdom; ^9^Department of Psychiatry, Experimental Biomedicine and Clinical Neuroscience (BIONEC), University of Palermo, Palermo, Italy

**Keywords:** schizophrenia, psychosis, outcomes, cardiovascular, health promotion intervention

## Abstract

**Introduction:** Cardiovascular morbidity and mortality are increased in severe mental illnesses (SMI). Trials of psychosocial health interventions to improve physical health in SMI, including in treatment-resistant schizophrenia, have shown some benefit. However, the representativeness of participants in such trials has not been determined.

**Method:** We utilized an anonymised case register to determine if participants in a randomized controlled trial (RCT) of a novel psychosocial health intervention aiming to improve physical health in SMI had similar severity of illness to eligible non-participants. A retrospective database analysis was performed, using Health of the Nation Outcome Scale (HoNOS) data from the sample of patients participating in the IMPaCT (Improving Physical health and reducing substance use in Psychosis) RCT (*n* = 293) compared to all eligible participants with a psychotic illness (*n* = 774).

**Results:** The mean total HoNOS score in the eligible comparator population (Mean = 9.09, *SD* = 5.8, range = 0–30) was significantly greater than that of the IMPaCT RCT participants (Mean = 7.16, SD = 4.7, range = 0–26), (*t* = 3.810, *p* = 0.006), as was the degree of overall illness severity and functional impairment, as measured by HoNOS.

**Conclusion:** This study shows for the first time that the patient population participating in an RCT of a lifestyle intervention for those with SMI had a better mental health status at entry to the trial, than the total eligible population, although there was no difference in physical health needs. This has relevance to the applicability of RCTs of lifestyle interventions in service planning and suggests that when people are more unwell, greater effort may be needed to include them in psychosocial interventions. A more careful and focused recruitment approach should be followed to improve the participation of the more severely ill patients in psychosocial interventions in order to enhance the external validity of such studies.

## Introduction

People with severe mental illness (SMI), such as schizophrenia, schizoaffective disorder and bipolar affective disorder, have reduced life expectancy compared to those in the general population (1–3). Most of this excess mortality is due to physical illnesses, with cardiovascular disease prominent (4, 5). Meta-analyses have demonstrated that targeted behavioral and non-specific psychosocial interventions can be beneficial in reducing antipsychotic induced weight gain and improving metabolic parameters (6–8). However, in order to determine the external validity of these findings (i.e., the extent to which the results can be generalized to clinical practice), we must determine how the extent to which participants in such studies are representative of people with SMI.

Similar concerns have been raised in relation to inclusion criteria for RCTs investigating the efficacy of clozapine in treatment resistant schizophrenia (TRS), where a broader range of patients are included than those meeting strict criteria for treatment resistance, with inclusion of treatment intolerant and non-refractory cases (9, 10).

Although there are examples of studies that have followed up non-randomized patients in clinical trials making it possible to assess and describe the generalizability of the results (11), few studies involving people with SMI have compared the characteristics of participating and non-participating patients, thus limiting the external validity of study findings. One difficulty in doing so lies in the ethical challenges of obtaining clinical data pertaining to individuals who have not consented to participate in the study (12, 13). The generalisability and real world translation of research in SMI is further limited by non-participation which may be selective (13). For example, at the more severe spectrum of mental illness, people may lack the capacity to consent to research, reducing the representativeness of research samples (14). Participation rates for studies in SMI are thought to be low, although this has not been as widely documented as the high dropout rates in this population (15). A recent survey of a large representative sample of people with psychotic disorders identified that 65% (*n* = 773) of those approached consented to participate in research, with older people less likely to do so (16).

Obstacles to participation include illness severity (17–19), fear of worsening of mental state due to participation (20), and concerns about adverse treatment effects (21). Negative symptomatology in patients with schizophrenia, with its inherent poor motivation and communication difficulties, may further reduce participation and limit the applicability of study findings (13).

While there is extensive work aimed at improving the participation of eligible patients (22) and identifying barriers to patient participation in mental health research (23), little is known about the clinical characteristics of non-participants. In particular, no such data exists for studies of non-pharmacological interventions to improve metabolic parameters in SMI, an important gap in the evidence on which to plan services.

We set out to determine the clinical characteristics of patients eligible to participate in the Improving Physical health and reducing substance use in Psychosis (IMPaCT) randomized controlled trial (RCT) (24) and compare these to the participating group using the Health of the Nations Outcome Scale (HoNOS) scores (25). We hypothesized that overall, individuals who were eligible to participate in the RCT would be more severely ill and functionally impaired as assessed by Health of the Nations Outcome Scale (HoNOS) scores (25), compared to those who agreed to participate.

## Materials and Methods

This current study is a secondary analysis of the Improving Physical health and reducing substance use in Psychosis (IMPaCT) cluster randomized controlled trial (RCT) (Trial registration: ISRCTN58667926) (24, 26). The IMPaCT study is a multicentre, two arm, parallel cluster randomized controlled trial (RCT) of a psychosocial health promotion intervention (IMPACT Therapy) in people with a diagnosis of SMI (24). The patient-tailored IMPACT Therapy aimed to target one or more health behaviors from a pre-defined list that includes cannabis use; alcohol use; other substance use; cigarette smoking; exercise; diet and diabetic control, prioritizing those identified as problematic by the patient, taking a motivational interviewing (MI) and cognitive behavioral therapy (CBT) approach. Participants were permitted to start the community-based IMPACT Therapy as soon as they were well-enough to attend, even if they were in-patients, to mirror clinical practice (24, 26).

The aim of this current study is to determine whether participants in the IMPaCT RCT were representative of the target population in respect of levels of illness severity and functional impairment.

### Setting

The South London and Maudsley NHS Foundation Trust (SLaM) is one of the largest providers of secondary mental health care in Europe. It provides mental healthcare across four London boroughs (Croydon, Lambeth, Lewisham, and Southwark) to a population of 1.2 million. Since 2006, electronic records have been used across all SLAM services. Since 2008 the Case Register Interactive Search (CRIS) system has been developed to allow for the search and retrieval of anonymised electronic clinical records of patient data (27). Over 250,000 cases are currently represented on CRIS in the form of detailed anonymised clinical information (28). The protocol for this case register has been described in an open-access publication (27).

### Participants and Recruitment

In the IMPaCT RCT, to maximize inclusiveness, care coordinators from continuing care/recovery teams, community rehabilitation, assertive outreach, and community forensic teams were recruited in random order and the eligible patients of consenting care co-ordinators likewise approached for inclusion in the study in random order. The inclusion criteria for IMPaCT service user participants were as follows: male or female aged between 18 and 65 years old; community patients with a primary diagnosis of a non-affective psychotic disorder (ICD10 diagnostic criteria: F20.0–F29.0) or an affective psychotic disorder (F30–33). Exclusion criteria included a primary diagnosis of learning disability; a first episode of psychosis; serious physical illness that could impact metabolic measures and substance misuse; pregnant or up to 6 months post-partum; or receiving intensive care for a medical or terminal condition.

In this present study, the comparator population comprised the patients meeting the same inclusion criteria who were on the overall caseload of each of the participating care coordinators. The IMPaCT group were therefore a subset of the comparator population.

Individuals on the comparator caseload were included in the analysis if they had been assessed by a mental health professional using the Health of the Nations Outcome Scale (HoNOS) at least once in a time period of plus or minus 6 months from the date of the recruitment of the care coordinator to the IMPaCT RCT. If the individual had required an acute psychiatric hospital admission during that period they were excluded from the comparator population. Further, if no HoNOS score was completed in the 12 months study period, then the patient was excluded.

### Outcome Measures

The primary measure of interest was the total HoNOS scale score, as this was routinely collected clinically and so was available in the pseudoanonymised clinical sample as well as the research sample. We compared HoNOS in patients participating in the IMPaCT RCT compared to that of all patients with SMI in the caseload of their care coordinators. Sociodemographic characteristics were not taken for comparison, as this would have potentially identified study non-participants.

The HoNOS has 12 items and four sections measuring, respectively: behaviors, impairments, symptoms, and social functioning (25).

A total score from the 12 items gives a measure of illness severity. Individual items and subscales in the 4 domains can be analyzed to assess their relative contributions to global functioning. Each item is measured on a 5-point Likert scale where; 0 = no problem within the period rated, 1 = sub-threshold problem, 2 = mild but definitely present, 3 = moderately severe, and 4 = severe to very severe, making 48 the highest possible score on the HoNOS (29).

Individuals in the total caseload who had a HoNOS score assessed during the 12 months study period were included. In the analysis we used the earliest HoNOS completed closest to the recruitment date of the relevant care coordinator to the RCT. HoNOS scores for individuals who had a hospitalization or an admission to a high intensity community support team [such as a Home Treatment Team (HTT)] within 6 months before or after the recruitment date were excluded to reduce the likelihood of us merely demonstrating that a short-term deterioration in mental state reduces participation in community-based trials. We did not exclude participants in the IMPaCT study group who were admitted to hospital or to a Home Treatment Team (HTT) following recruitment to the trial, although trial recruitment solely took place in the community.

The HoNOS scores of interest for this analysis were total HoNOS scale scores, subscale scores and individual itemized HoNOS scale scores. Items 9, 10, 11, and 12 in particular were assessed as a measure of functional impairment.

The means of total HoNOS scores from the overall care coordinator caseload and IMPaCT RCT participants were used as a proxy measure of global illness severity. Secondary outcomes were the “functional impairment” HoNOS subscale, which was composed of: item 9, “impairments in interpersonal relationships” (such as social withdrawal); item 10- “impairments in activities of daily living” (such as washing, dressing, mobility, and use of transport); item 11- “deficits in the quality of living conditions” (including absence of basic necessities such as heat and light); and item 12- “impairments in occupational functioning” (including the ability to engage in occupational and recreational activities).

In the study, HoNOS ratings were conducted by mental health professionals directly involved in the care of patients care for the IMPaCT non-participants and by clinical researchers for the IMPaCT study participants. It was not possible to measure interrater reliability for HoNOS scores between the clinical staff and the IMPaCT clinical researchers.

### Statistical Analysis

For this analysis, a paired-samples *t*-test was used to compare:

1. total HoNOS scores from the overall caseload data set and from the IMPaCT RCT participant data set
2. HoNOS subscale scores (items 9, 10, 11, and 12) in the overall caseload data set and the IMPaCT RCT participant data set
3. individual HoNOS item scores between the overall caseload group and the IMPaCT RCT participants group.

The statistical package SPSS version 24 was used for the analyses and all *t*-tests were two-tailed with statistical significance set at an alpha level of *p* ≤ 0.05.

## Results

The IMPaCT RCT recruited 293 eligible participants from within SLaM, who were on the caseload of 68 care coordinators. In this present study, using CRIS, we identified on the overall caseload of each of the 68 eligible care coordinators, a total comparator population comprising 1,109 patients who met the inclusion criteria, including having a primary diagnosis of a psychotic disorder. Of these 1,109 patients, 19 were excluded due to incomplete HoNOS scoring in the 12 month period from the time of the RCT recruitment, giving a HoNOS completion rate of 98.3%, and leaving a total of 1,090 in the comparator group. A further 316 (21.7% of those with a psychotic disorder) were excluded having had a hospitalization or admission to a HTT over the study period, leaving 774 patients in the comparator group for analysis.

The mean total HoNOS score in the target population (*n* = 774) (Mean = 9.09, *SD* = 5.8, range = 0–30) was significantly greater than the mean total HoNOS score for the IMPaCT RCT participating group (*n* = 293) (Mean = 7.16, *SD* = 4.7, range = 0–26), (*t* = 3.810, *p* < 0.001). Comparison of the individual HoNOS item scores between those participating in the RCT and those on the care coordinators total caseload is shown in Table 1.

**Table 1:** Comparison of Mean HoNOS item scores between total caseload and RCT participants.

**HoNOS scale item**	**Mean HoNOS item scores for Comparator group (*n* = 293)**	**Mean HoNOS item scores for IMPaCT group (*n* = 774)**	**Mean difference (SD) between total caseload and IMPaCT participants**	***T*-test; *p***
Overactivity and aggression	0.49	0.33	0.16 (0.07)	2.284; 0.023^*^
Non-Accidental self-injury	0.10	0.09	0.01 (0.04)	0.262; 0.794
Problem drinking or drug-taking	0.32	0.33	−0.005 (0.07)	−0.070; 0.945
Cognitive problems	0.74	0.53	0.215 (0.08)	2.855; 0.005^*^
Physical illness or disability problems	0.92	0.78	0.14 (0.01)	−1.280; 0.201
Hallucinations and delusions	1.22	1.06	0.17 (0.11)	−1.134; 0.258
Depressed mood	0.72	0.71	−0.10 (0.08)	1.469; 0.143
Other symptoms (not delusions or hallucinations)	1.25	0.95	0.29 (0.10)	−2.787; 0.006^*^
Problems with relationships	1.08	0.78	0.30 (0.09)	−3.388; 0.001^*^
Problems with Activities of daily living	1.07	0.74	0.34 (0.10)	−3.041; 0.001^*^
Problems with living conditions	0.74	0.45	0.14 (0.08)	4.225; 0.003^*^
Problems with occupation and activities	0.75	0.60	0.16(0.10)	−1.641; 0.102
Total HoNOS caseload-Total HoNOS IMPaCT	9.09	7.16	1.93 (0.51)	3.810; 0.001^*^
HoNOS functional impairment (items 9,10,11, and 12) caseload-HoNOS functional impairment IMPaCT	3.35	2.41	0.94 (0.24)	3.945; 0.001^*^

* *p < 0.05*.

There were significantly increased scores for HoNOS item 1 (overactive, aggressive, or agitated behavior regardless of cause), item 4 (cognitive problems), item 8 (symptoms due to other mental or behavioral problems), item 9 (problems with relationships), item 10 (problems with activities of daily living), and item 11 (problems with living conditions) in the comparator group when compared to the IMPaCT participants (see Table 1). There was no significant difference in the HoNOS scores on the physical health item between the comparator group and the IMPaCT study group [Mean difference (MD) = 0.24; *t* = 1.408, *p* = 0.161].

There was a significant increase in the HoNOS subgroup score for items 9,10,11, and 12 in the comparator group [mean HoNOS score for items 9,10,11, and 12 = 3.35 (*SD* = 2.6)] compared to the IMPaCT group [mean HoNOS score for items 9,10,11, and 12 = 2.41 (*SD* = 2.3) (*t* = 3.945, *p* < 0.001]. This indicates that the comparator group were more functionally impaired than the IMPaCT study participants. The frequency of responses to each of the HoNOS items by group is shown in Table 2.

**Table 2:** Cohort characteristics itemized by HoNOS scale items.

**HoNOS items**	**Total caseload (*n* = 776) *N* = (%)**	**IMPaCT participants (*n* = 293) *N* = (%)**
**OVERACTIVITY AND AGGRESSION**		
Not a problem	539 (70)	223 (76)
Subclinical, minor problems requiring no action	153 (20)	53 (18)
Mild to very severe problem	84 (10)	23 (6)
**NON-ACCIDENTAL SELF-INJURY**		
Not a problem	721 (93)	268 (91)
Subclinical, minor problems requiring no action	46 (6)	26 (8.7)
Mild to very severe problem	9 (1)	1(0.3)
**PROBLEM DRINKING OR DRUG-TAKING**		
Not a problem	585 (76)	223 (76)
Subclinical, minor problems requiring no action	111 (14)	44 (15)
Mild to very severe problem	79 (10)	28 (9)
**COGNITIVE PROBLEMS**		
Not a problem	416 (54)	166 (56)
Subclinical, minor problems requiring no action	239 (31)	100 (34)
Mild to very severe problem	121 (15)	29 (10)
**PHYSICAL ILLNESS OR DISABILITY PROBLEMS**		
Not a problem	357 (46)	155 (52)
Subclinical, minor problems requiring no action	192 (25)	64 (22)
Mild to very severe problem	227 (29)	76 (26)
**HALLUCINATIONS AND DELUSIONS**		
Not a problem	314 (41)	127 (43)
Subclinical, minor problems requiring no action	171 (22)	47 (16)
Mild to very severe problem	290 (37)	121 (41)
**DEPRESSED MOOD**		
Not a problem	394 (51)	147 (50)
Subclinical, minor problems requiring no action	264 (34)	96 (33)
Mild to very severe problem	118 (15)	52 (17)
**OTHER SYMPTOMS**		
Not a problem	94 (24)	124 (42)
Subclinical, minor problems requiring no action	291 (38)	73 (25)
Mild to very severe problem	90 (38)	98 (33)
**PROBLEMS WITH RELATIONSHIPS**		
Not a problem	79 (36)	136 (46)
Subclinical, minor problems requiring no action	266 (35)	89 (30)
Mild to very severe problem	237 (29)	70 (24)
**PROBLEMS WITH ACTIVITIES OF DAILY LIVING**		
Not a problem	338 (44)	161 (55)
Subclinical, minor problems requiring no action	176 (23)	69 (23)
Mild to very severe problem	261 (33)	65 (22)
**PROBLEMS WITH LIVING CONDITIONS**		
Not a problem	545 (71)	221 (75)
Subclinical, minor problems requiring no action	131 (17)	53 (18)
Mild to very severe problem	97 (12)	19 (7)
**PROBLEMS WITH OCCUPATION AND ACTIVITIES**		
Not a problem	396 (51)	191 (65)
Subclinical, minor problems requiring no action	193 (25)	55 (19)
Mild to very severe problem	183 (24)	48 (16)

## Discussion

To the best our knowledge, this is the first time that comparative levels of illness severity and functional impairment in a large, eligible non-participating group, and participating sample of a psychosocial health intervention RCT in SMI have been explored. This study demonstrates that both illness severity and functional impairment were increased in the non-participating population compared to the participating group. The overall health status was better in the study population, and the less severely ill patients were recruited to this trial.

Of interest, the levels of physical health problems were similar between both groups. This is relevant, as the IMPaCT trial intervention was designed to effect physical health improvements in the study population. The comparability between physical health impairment in both groups suggests that this did not drive participation selection bias, as would have been indicated by either increased severity of physical illness in the non-participating comparator group or indeed, by people with more physical health problems electing to participate. Instead, it appears there was an equivalent physical health need, but that other factors accounted for the difference in uptake of the research opportunity.

Limitations of this study need to be considered. It was not possible to assess for the effect of gender, age, ethnicity, and duration of illness of the participating and non-participating patient populations. Our inability to investigate factors that may be predictive of non-trial participation is a limitation, information that would be informative to improve the design of future RCTs. Studies have indicated that older age (16, 23) and ethnicity (30), specifically black ethnicity (16), are barriers to recruitment in mental health studies, factors which may be related to illness severity. Clinical data that may have impacted on study involvement, such as duration of illness and number of psychiatric hospitalizations, were not available in the pseudoanonymised comparator sample. However, the two study populations were comparable on clinical symptoms such as hallucinations/delusions, and depression, indicating that active symptoms of mental illness were not impacting on the participation rates between the groups. Data were obtained retrospectively, which may limit the generalisability of the findings. However, the recruitment of a patient population for a prospective study would be difficult, and the method used has enabled us to for the first time compare these groups in a behavioral intervention in SMI.

In the analysis, we used only a single HoNOS score based on the first HoNOS assessment in the relevant study period. This precludes a more encompassing assessment of fluctuating symptom profiles over time. The use of the HoNOS provides a behavioral assessment of functioning at the level of individual items, but does not allow for assessment of discrepancies between behavior and inner experience. It may be that in a population of individuals with SMI that the significantly increased functional impairment in the inclusive comparator group is related to negative symptomatology (31, 32), but this is something that we were not able to assess.

In this study, HoNOS scores measured by care coordinators for the comparator population were compared with those measured by the IMPaCT RCT researchers. HoNOS is reported to show a moderate inter-rater reliability, but this is improved with training in HoNOS completion (33). Both care coordinators and researchers in the IMPaCT RCT study were trained in the use of the HoNOS, with the expectation that this would support inter-rater reliability.

Strengths of this study include the size and comprehensiveness of the sample. We were able to access the clinical records of over 1,000 community dwelling individuals with psychotic disorders. We looked at data specifically relating to individuals with SMI living in the community and purposefully excluded those who were hospitalized over the study period. This aids the applicability of our study findings to ambulatory research and enhances the generalisability of the study findings. Due to the comprehensiveness of the search tool, we were able to identify a patient population that reflects the characteristics of patients seen in standard clinical practice thus further enhancing the validity of our findings.

An additional important finding in this study was the high rate of HoNOS completion in the sample of community dwelling patients with SMI (98% completion rate). This finding demonstrates the clinical utility of the HoNOS and a high level of acceptability for its use in community mental health settings, although this was likely enhanced in UK practice by HoNOS measures being used for funding models. These findings are however mirrored in other community samples, such as in New Zealand where high completion rates of the HoNOS are documented, more so than in inpatient settings (95 and 79% respectively) (34).

The greater illness severity in the comparator group as compared to the participating group is unlikely to be a result of the recruitment and randomization method in the IMPaCT RCT. An important recruitment factor in the IMPaCT RCT was the use of relatively wide inclusion criteria, including dual diagnosis, and complex patients.

We cannot tell from these data whether the more ill patients would have participated in the psychosocial health promotion intervention were it not part of a clinical trial. If research itself were the barrier, it raises questions as to whether published research into psychosocial interventions for physical health is applicable to the patients with the greatest impairment in health and social functioning. This mirrors more broad concerns regarding the representativeness of RCTs of treatment interventions in SMI, and how this impacts on translation to real world clinical practice. Observational data can help in this regard. Clinical implementation trials for physical health problems in SMI are required (35), which may be pragmatic large scale trials to ensure broad inclusion criteria, heterogeneous populations and assessment of the real world effectiveness of psychosocial interventions for physical health (36). There remains limited data on what factors predict entry to psychosocial intervention trials in SMI. An increased awareness of this may aid increased knowledge of when medication based and/or psychosocial interventions are preferable to psychosocial interventions alone.

Our findings have implications for future RCTs of psychosocial intervention in SMI. The study finding that individuals with greater functional impairment are less likely to participate in RCTs should lead to focused interventions to increase their participation. This is required in order to ensure that trial results can be confidently translated into clinical practice.

## Author Contributions

JL and FG conceptualized the study. JL, SS, RM, and FG contributed to the design. JL, RW, SN, and HS conducted the data collection. JL and RW undertook the statistical analysis. JL wrote the first draft of the manuscript. All authors contributed to and have approved the final manuscript.

### Conflict of Interest Statement

RM has received honoraria for lectures from Lundbeck, Otsuka, Janssen, and Sunovian. FG has received honoraria for advisory work and lectures or CME activity support from Roche, BMS, Lundbeck, Otsuka, Janssen, and Sunovion, is a collaborator on a NHS Innovations project co-funded by Janssen and has a family member with professional links to Lilly and GSK, including shares. The remaining authors declare that the research was conducted in the absence of any commercial or financial relationships that could be construed as a potential conflict of interest.

## References

[1] Chang CK Hayes RD Perera G Broadbent MT Fernandes AC Lee WE. Life expectancy at birth for people with serious mental illness and other major disorders from a secondary mental health care case register in London. PLoS ONE (2011) 6:e19590. 10.1371/journal.pone.0019590 21611123 PMC3097201

[2] Oakley P Kisely S Baxter A Harris M Desoe J Dziouba A. Increased mortality among people with schizophrenia and other non-affective psychotic disorders in the community: a systematic review and meta-analysis. J Psychiatr Res. (2018) 102:245–53. 10.1016/j.jpsychires.2018.04.019 29723811

[3] Walker E McGee RE Druss BG. Mortality in mental disorders and global disease burden implications: a systematic review and meta-analysis. JAMA Psychiatry (2015) 72:334–41. 10.1001/jamapsychiatry.2014.2502 25671328 PMC4461039

[4] Correll CU Solmi M Veronese N Bortolato B Rosson S Santonastaso P. Prevalence, incidence and mortality from cardiovascular disease in patients with pooled and specific severe mental illness: a large-scale meta-analysis of 3,211,768 patients and 113,383,368 controls. World Psychiatry (2017) 16:163–80. 10.1002/wps.20420 28498599 PMC5428179

[5] Westman J Eriksson SV Gissler M Hallgren J Prieto ML Bobo WV. Increased cardiovascular mortality in people with schizophrenia: a 24-year national register study. Epidemiol Psychiatr Sci. (2017) 27:519–27. 10.1017/S2045796017000166 28580898 PMC6137375

[6] Caemmerer J Correll CU Maayan L. Acute and maintenance effects of non-pharmacologic interventions for antipsychotic associated weight gain and metabolic abnormalities: a meta-analytic comparison of randomized controlled trials. Schizophr Res. (2012) 140:159–68. 10.1016/j.schres.2012.03.017 22763424 PMC10416544

[7] McGinty EE Baller J Azrin ST Juliano-Bult D Daumit GL. Interventions to address medical conditions and health-risk behaviors among persons with serious mental illness: a comprehensive review. Schizophr Bull. (2016) 42:96–124. 10.1093/schbul/sbv101 26221050 PMC4681556

[8] Taylor J Stubbs B Hewitt C Ajjan RA Alderson SL Gilbody S. The effectiveness of pharmacological and non-pharmacological interventions for improving glycaemic control in adults with severe mental illness: a systematic review and meta-analysis. PLoS ONE (2017) 12:e0168549. 10.1371/journal.pone.0168549 28056018 PMC5215855

[9] Lally J Ajnakina O Di Forti M Trotta A Demjaha A Kolliakou A. Two distinct patterns of treatment resistance: clinical predictors of treatment resistance in first-episode schizophrenia spectrum psychoses. Psychol Med. (2016) 46:3231–40. 10.1017/S0033291716002014 27605254

[10] Taylor DM. Clozapine for treatment-resistant schizophrenia: still the gold standard? CNS Drugs (2017) 31:177–80. 10.1007/s40263-017-0411-6 28258419

[11] Rovers MM Straatman H Ingels K van der Wilt GJ van den Broek P Zielhuis GA Generalizability of trial results based on randomized versus nonrandomized allocation of OME infants to ventilation tubes or watchful waiting. J Clin Epidemiol. (2001) 54:789–94. 10.1016/S0895-4356(01)00340-7 11470387

[12] Haapea M Miettunen J Veijola J Lauronen E Tanskanen P Isohanni M. Non-participation may bias the results of a psychiatric survey: an analysis from the survey including magnetic resonance imaging within the Northern Finland 1966 Birth Cohort. Soc Psychiatry Psychiatr Epidemiol. (2007) 42:403–9. 10.1007/s00127-007-0178-z 17404677

[13] Riedel M Strassnig M Müller N Zwack P Möller HJ. How representative of everyday clinical populations are schizophrenia patients enrolled in clinical trials? Eur Arch Psychiatr Clin Neurosci. (2005) 255:143–8. 10.1007/s00406-004-0547-5 15549345

[14] Whelan PJ Walwyn R Gaughran F Macdonald A. Impact of the demand for ‘proxy assent’ on recruitment to a randomised controlled trial of vaccination testing in care homes. J Med Ethics (2013) 39:36–40. 10.1136/medethics-2011-100119 22942376

[15] Leucht S Heres S Hamann J Kane JM. Methodological issues in current antipsychotic drug trials. Schizoph Bull. (2008) 34:275–85. 10.1093/schbul/sbm159 18234700 PMC2632403

[16] Patel R Oduola S Callard F Wykes T Broadbent M Stewart R. What proportion of patients with psychosis is willing to take part in research? A mental health electronic case register analysis. BMJ Open (2017) 7:e013113. 10.1136/bmjopen-2016-013113 28279995 PMC5353309

[17] Bowen J Hirsch S Recruitment rates and factors affecting recruitment for a clinical trial of a putative anti-psychotic agent in the treatment of acute schizophrenia. Human Psychopharmacol. (1992) 7:337–41. 10.1002/hup.470070507

[18] Fischer EH Dornelas EA Goethe JW. Characteristics of people lost to attrition in psychiatric follow-up studies. J Nerv Ment Dis. (2001) 189:49–55. 10.1097/00005053-200101000-00009 11206665

[19] Hofer A Hummer M Huber R Kurz M Walch T Fleischhacker W. Selection bias in clinical trials with antipsychotics. J Clin Psychopharmacol. (2000) 20:699–702. 10.1097/00004714-200012000-00019 11106145

[20] Kaminsky A Roberts LW Brody JL. Influences upon willingness to participate in schizophrenia research: an analysis of narrative data from 63 people with schizophrenia. Ethics Behav. (2003) 13:279–302. 10.1207/S15327019EB1303_06 14680009

[21] Zullino D Conus P Borgeat F Bonsack C. Readiness to participate in psychiatric research. Can J Psychiatry (2003) 48:480–4. 10.1177/070674370304800709 12971019

[22] Treweek S Mitchell E Pitkethly M Cook J Kjeldstrom M Taskila T Strategies to improve recruitment to randomised controlled trials. Cochrane Database Syst Rev. (2010) 20:MR000013 10.1002/14651858.MR000013.pub5 20393971

[23] Woodall A Morgan C Sloan C Howard L. Barriers to participation in mental health research: are there specific gender, ethnicity and age related barriers? BMC Psychiatry (2010) 10:103. 10.1186/1471-244X-10-103 21126334 PMC3016310

[24] Gaughran F Stahl D Ismail K Greenwood K Atakan Z Gardner-Sood P. Randomised control trial of the effectiveness of an integrated psychosocial health promotion intervention aimed at improving health and reducing substance use in established psychosis (IMPaCT). BMC Psychiatry (2017) 17:413. 10.1186/s12888-017-1571-0 29284438 PMC5745644

[25] Wing JK Beevor AS Curtis RH Park SB Hadden S Burns A. Health of the nation outcome scales (HoNOS). Res Dev Br J Psychiatry (1998) 172:11–8. 10.1192/bjp.172.1.11 9534825

[26] Gaughran F Stahl D Ismail K Atakan Z Lally J Gardner-Sood P. Improving physical health and reducing substance use in psychosis—randomised control trial (IMPACT RCT): study protocol for a cluster randomised controlled trial. BMC Psychiatry (2013) 13:263. 10.1186/1471-244X-13-263 24131496 PMC3852764

[27] Stewart R Soremekun M Perera G Broadbent M Callard F Denis M. The south london and maudsley NHS foundation trust biomedical research centre (SLAM BRC) case register: development and descriptive data. BMC Psychiatry (2009) 9:9–51. 10.1186/1471-244X-9-51 19674459 PMC2736946

[28] Perera G Broadbent M Callard F Chang CK Downs J Dutta R. Cohort profile of the south london and maudsley NHS foundation trust biomedical research centre (SLaM BRC) case register: current status and recent enhancement of an electronic mental health record-derived data resource. BMJ Open (2016) 6:e008721. 10.1136/bmjopen-2015-008721 26932138 PMC4785292

[29] Orrell M Yard P Handysides J Schapira R. Validity and reliability of the health of the nation outcome scales in psychiatric patients in the community. Br J Psychiatry (1999) 174:409–12. 10.1192/bjp.174.5.409 10616606

[30] Jackson JS Torres M Caldwell CH Neighbors HW Nesse RM Taylor RJ. The national survey of american life: a study of racial, ethnic and cultural influences on mental disorders and mental health. Int J Methods Psychiatr Res. (2004) 13:196–207. 10.1002/mpr.177 15719528 PMC6878295

[31] Rabinowitz J Levine SZ Garibaldi G Bugarski-Kirola D Berardo CG Kapur S. Negative symptoms have greater impact on functioning than positive symptoms in schizophrenia: analysis of CATIE data. Schizophr Res. (2012) 137:147–50. 10.1016/j.schres.2012.01.015 22316568

[32] Ventura J Hellemann GS Thames AD Koellner V Nuechterlein KH. Symptoms as mediators of the relationship between neurocognition and functional outcome in schizophrenia: a meta-analysis. Schizophr Res. (2009) 113:189–99. 10.1016/j.schres.2009.03.035 19628375 PMC2825750

[33] Amin S Singh SP Croudace T Jones P Medley I Harrison G. Evaluating the health of the nation outcome scales. Reliability and validity in a three-year follow-up of first-onset psychosis. Br J Psychiatry (1999) 174:399–403. 10.1192/bjp.174.5.399 10616604

[34] Eagar K Trauer T Mellsop G. Performance of routine outcome measures in adult mental health care. Aust N Z J Psychiatry (2005) 39:713–8. 10.1080/j.1440-1614.2005.01655.x 16050925

[35] Lally J Stubbs B Guerandel A O'Shea D Gaughran F. Pharmacological management of diabetes in severe mental illness: a comprehensive clinical review of efficacy, safety and tolerability. Exp Rev Clin Pharmacol. (2018) 11:411–24. 10.1080/17512433.2018.1445968 29480037

[36] Stroup TS Geddes JR. Randomized controlled trials for schizophrenia: study designs targeted to distinct goals. Schizoph Bull. (2008) 34:266–74. 10.1093/schbul/sbm156 18245060 PMC2632397

